# Apolipoprotein E (ApoE) ε4 Genotype (ApoE rs429358—ApoE rs7412 Polymorphisms) Is Not Associated with Long COVID Symptoms in Previously Hospitalized COVID-19 Survivors

**DOI:** 10.3390/genes14071420

**Published:** 2023-07-10

**Authors:** César Fernández-de-las-Peñas, Lars Arendt-Nielsen, Gema Díaz-Gil, Francisco Gómez-Esquer, Antonio Gil-Crujera, Stella M. Gómez-Sánchez, Silvia Ambite-Quesada, María A. Palomar-Gallego, Oscar J. Pellicer-Valero, Rocco Giordano

**Affiliations:** 1 Department of Physical Therapy, Occupational Therapy, Rehabilitation and Physical Medicine, Universidad Rey Juan Carlos, 28922 Alcorcón, Spain; silvia.ambite.quesada@urjc.es; 2 Center for Neuroplasticity and Pain (CNAP), SMI, Department of Health Science and Technology, Faculty of Medicine, Aalborg University, DK-9220 Aalborg, Denmark; lan@hst.aau.dk (L.A.-N.); rg@hst.aau.dk (R.G.); 3 Department of Medical Gastroenterology, Mech-Sense, Aalborg University Hospital, DK-9000 Aalborg, Denmark; 4 Research Group GAMDES, Department of Basic Health Sciences, Universidad Rey Juan Carlos (URJC), 28922 Madrid, Spain; gema.diaz@urjc.es (G.D.-G.); francisco.gomez.esquer@urjc.es (F.G.-E.); antonio.gil@urjc.es (A.G.-C.); stella.gomez@urjc.es (S.M.G.-S.); mariaangustias.palomar@urjc.es (M.A.P.-G.); 5 Image Processing Laboratory (IPL), Universitat de València, Parc Científic, Paterna, 46100 València, Spain; oscar.pellicer@uv.es

**Keywords:** single nucleotide polymorphism, ApoE, genotype, long COVID

## Abstract

The role of genetics as a predisposing factor related to an increased risk of developing long COVID symptomatology is under debate. The aim of the current secondary analysis was to identify the association between the Apolipoprotein E (ApoE) gene, a gene affecting cholesterol metabolism and previously associated with a higher risk of SARS-CoV-2 infection and COVID-19 severity, and the development of long COVID in a cohort of individuals who had been hospitalized by SARS-CoV-2 infection. Unstimulated whole saliva samples were collected from 287 previously hospitalized COVID-19 survivors. Three genotypes of the ApoE gene (ApoE ε2, ε3, ε4) were obtained based on the combination of ApoE rs429358 and ApoE rs7412 polymorphisms. Participants were asked to self-report the presence of any post-COVID symptom in a face-to-face interview at 17.8 ± 5.2 months after hospital discharge and medical records were obtained. Each participant reported 3.0 (1.9) post-COVID symptoms. Overall, no significant differences in long COVID symptoms were observed depending on the ApoE genotype (ApoE ε2, ApoE ε3, ApoE ε4). The presence of the ApoE ε4 genotype, albeit associated with a higher risk of SARS-CoV-2 infection and COVID-19 severity, did not appear to predispose for the presence of long COVID in our cohort of previously hospitalized COVID-19 survivors.

## 1. Introduction

After billions of infections and millions of deaths worldwide, the coronavirus disease 2019 (COVID-19), caused by severe acute respiratory syndrome coronavirus 2 (SARS-CoV-2), has provoked the biggest humanitarian crisis of the current century. Viral mechanisms of infection of the SARS-CoV-2 suggest that genetic factors play an essential role in determining host responses [1]. Genetic variations, e.g., single nucleotide polymorphisms (SNPs), have been shown to be involved at different levels in the process of viral infection, affecting different genes implicated in processes regulating viral pathogenesis and the immune response pathways initiated by the virus in the infected host. Several studies have identified genes involved in the entry pathway of the SARS-CoV-2 virus, such as the surface receptor of the angiotensin-converting enzyme 2 (ACE2) and the transmembrane protease serine-2 (TMPRSS2) receptor. Other studies have focussed on those SNPs related to increased infection risk. In the latter scenario, the apolipoprotein E (ApoE) gene has been found to be a candidate for increased infection risk [2].

The ApoE gene transcribes for a protein that, though the association with fat brings about the formation of a lipoprotein, is responsible, in part, for removing cholesterol from the bloodstream. Variations in the ApoE gene affect cholesterol metabolism, and evidence shows that this alteration could lead to an increased possibility of having cardiac problems and other neurological diseases, e.g., Alzheimer’s [3]. There are three common allelic variants of the ApoE gene (ApoE-ε2, ApoE-ε3, ApoE-ε4), usually defined by two single nucleotide polymorphisms (SNPs), the rs429358 and the rs7412. A meta-analysis found that the ApoE ε4 genotype was associated with a higher risk of SARS-CoV-2 infection and with severe COVID-19 disease [4]. In fact, this association between the ApoE ε4 genotype and the severity of COVID-19 was not associated with the presence of underlying diseases predisposing to severe COVID-19 [5]. One possible explanation is that the elevated cholesterol and oxidized lipoprotein levels associated with the effects of the ApoE ε4 genotype are associated with increased pneumocyte susceptibility to infection and lung inflammation [6]. Another hypothesis points towards a possible negative regulation of ACE2 protein expression by the ApoE ε4 genotype [7], and ACE2 is essential for the SARS-CoV-2 virus to enter lung tissue.

Based on these potential mechanisms, it could be hypothesized that the ApoE gene may be associated with the presence of symptoms after the acute phase of the infection. The presence of symptoms persisting after the acute phase of SARS-CoV-2 infection has been called long COVID [8], although no consensus exists. Soriano et al., following the Delphi design, proposed the term “post-COVID-19 condition” and the following consensus definition: 

“Post-COVID-19 condition occurs in people with a history of probable or confirmed SARS-CoV-2 infection, usually three months from the onset of COVID-19 disease with symptoms that last for at least two months and cannot be explained by an alternative medical diagnosis” [9]. Our research group has recently reported that different SNPs related to the acute phase of SARS-CoV-2 infection and a higher risk of COVID-19 severity, specifically, ACE2 rs2285666, ACE2 rs2074192, TMPRSS2 rs12329760, TMPRSS2 rs2070788, and ACE1 rs1799752, do not predispose for long COVID symptomatology in previously hospitalized COVID-19 survivors [10,11]. 

We present a secondary analysis of this genetic part of THE LONG COVID EXPERIENCE STUDY [10,11] analyzing the potential association between a metabolic gene, such as the ApoE gene (based on ApoE rs429358-ApoE rs7412 polymorphisms), and a potential risk of developing long COVID symptoms. 

## 2. Methods

### 2.1. Participants

The recruitment process of the LONG-COVID-EXP study has been previously detailed [10]. Briefly, a sample of 287 individuals who had been previously hospitalized due to an acute SARS-CoV-2 infection during the first wave of the COVID-19 outbreak (March–May 2020) in three urban hospitals in Madrid (Spain) participated. The diagnosis of SARS-CoV-2 should have been conducted at hospital admission by real-time reverse transcription-polymerase chain reaction (RT-PCR) assay of nasopharyngeal and oral swab samples. The study was approved by the Institutional Ethics Committees of all participating institutions (HSO25112020; URJC0907202015920; HUFA20/126; HUIL/092-20) and all the participants provided their written informed consent before any data were collected.

### 2.2. DNA Collection and Genotyping

The genotyping collection procedures have been previously published [10]. Briefly, unstimulated whole saliva samples were collected from each subject and centrifuged at 3000 rpm for 15 min to obtain the cell sediment, which was then stored at −20 °C. 

Genomic DNA was extracted from 500 mL of saliva using a MagMAX™ DNA Multi-Sample Ultra 2.0 Kit (Thermo Fisher Scientific Inc, Hemel Hempstead, Hertfordshire, UK). We extracted DNA using a King Fisher Flex purification robot (Thermo Fisher, Waltham, MA, USA). The purity and concentration of the resulting DNA were assessed with Quant-iT™ PicoGreen™ dsDNA reagent” (Thermo Fisher). The DNA was diluted to 5 ng/μL, using 1× Tris-EDTA (TE) buffer (Sigma-Aldrich, Dorset, UK). Each 10 uL qPCR reaction mixture contained a total of 10 ng gDNA as a PCR template, 1× TaqMan Gene Expression PCR Master Mix, and 0.6× Genotyping TaqMan-probe assay. 

The taqMan^®^ Predesigned SNP Genotyping Assay (Thermo Fisher Scientific Inc., Hertfordshire, UK) was used for genotyping the SNPs with the real-time PCR reaction (RT-PCR). Real-time PCR plates were run in the Quantstudio 12K Flex System (Thermo Fisher) of the Genomics Unit (Madrid Science Park Foundation, Madrid, Spain) under standard conditions (95 °C for 10 min and 40 two-step cycles consisting of 95 °C for 15 s and 60 °C for 1 min) and analyzed with the Genotyping App of Thermo Fisher Cloud. Identification of each of the possible variants of each SNP was conducted by using specific fluorescent dyes [10]. 

Identification of each genotype of the APOE rs429358 and APOE rs7412 polymorphisms was conducted by using specific fluorescent dyes. The three possible variants of APOE rs429358 lead to the following genotypes (C/C, C/T, T/T) derived from C→T substitution at the following sequence: 

GCTGGGCGCGGACATGGAGGACGTG[C/T]GCGGCCGCCTGGTGCAGTACCGCGG

Similarly, the three possible variants of APOE rs7412 SNP lead to the following genotypes (C/C, C/T, T/T) derived from C→T substitution at the following sequence: 

CCGCGATGCCGATGACCTGCAGAAG[C/T]GCCTGGCAGTGTACCAGGCCGGGGC

Each of the haplotypes of the ApoE gene (ε2: T/T, ε3: C/T, ε4: C/C) was generated from combinations of these two SNPs.

### 2.3. Collection Data

Demographic, medical comorbidity, and hospitalization data were collected from hospital medical records. A list of post-COVID symptoms was collected in a face-to-face interview conducted by experienced healthcare professionals at 17.8 (SD 5.2) months after hospitalization [10]. We defined a post-COVID symptom as any symptom starting no later than one month after the SARS-CoV-2 acute infection and persisting at the time of the study.

### 2.4. Statistical Analysis

Differences in genotype frequencies of each SNP were analyzed with χ^2^ tests for categorical variables or one-way ANOVA tests for continuous variables. The Shapiro–Wilk test was used to assess the assumption of normality. For all inferences, the level of significance was set at priori 0.05 with *p*-values from all tests being corrected (Holm–Bonferroni correction). 

## 3. Results

The recruitment process has been previously detailed [10]. The current secondary analysis showed that individuals carrying the ApoE ε4 genotype were younger than those carrying the ApoE ε2 and ApoE ε3 genotypes (*p* = 0.03). No significant differences in previous medical comorbidities were identified depending on the ApoE genotype (Table 1).

Each participant reported a mean of 3.0 (1.9) post-COVID symptoms. Dyspnea at exertion (*n* = 196, 68%) and fatigue (*n* = 181, 63%) were the most prevalent post-COVID symptoms in the total sample. The ApoE genotype distribution (ApoE ε2 *n* = 25, ApoE ε3 *n* = 204, ApoE ε4 *n* = 58) deviated from the Hardy–Weinberg equilibrium (*p* > 0.05), a result also commonly found in COVID-19 studies. Overall, no significant differences in the presence of post-COVID symptoms were observed based on the ApoE genotype (Table 1). In addition, no sex differences in the genotype distribution (*p* = 0.986) were found.

## 4. Discussion

This is the first report investigating the association between the ApoE ε4 genotype and the risk of developing long COVID symptoms. The results of this secondary analysis revealed that the ApoE ε4 genotype, albeit previously associated with a higher risk of infection and COVID-19 severity [4], does not predispose to long COVID symptomatology in the cohort evaluated of previously hospitalized COVID-19 survivors. The current results follow our previous reports on other SNPs associated with COVID-19 severity, such as the ACE2, TMPRSS2, and ACE1 polymorphisms [10,11], suggesting that long COVID seems not to be associated with COVID-19-associated polymorphisms. This lack of association would agree with recent results confirming that the severity of COVID-19 disease is also not a risk factor for developing long-lasting post-COVID symptoms [12].

Different hypotheses could explain the lack of association between ApoE genotype and long COVID symptomatology. First, the lack of association between host genetics and long COVID could be explained by a significant gene variability existing (i.e., ethnic differences) across populations [13]. This hypothesis would agree with the finding that the ApoE genotype distribution deviated from the Hardy–Weinberg equilibrium in the current sample. In fact, this is a finding commonly observed in COVID-19 studies due to various factors such as population stratification and genetic drift. Thus, genetic variability should be considered in genetic studies including COVID-19 survivors. Another explanation could be that those medical comorbidities associated with the ApoE genotype. In this vein, Lumsden et al. observed that the ApoE ε4 genotype is associated with a higher risk of hypercholesterolemia and ischemic heart disease but also with a lower risk (protection effect) of obesity, chronic airway obstruction, and type 2 diabetes, among others [14]. A recent systematic review identified obesity as a potential risk factor for long COVID [15]. Nevertheless, the association of other comorbidities with long COVID is heterogeneous, since hypertension has been found to be associated with a higher number of post-COVID symptoms [16], whereas diabetes was not [17]. In the current study, no differences in previous medical comorbidities, particularly those associated with COVID-19 severity such as obesity, hypertension, or diabetes, based on the ApoE genotype were observed, potentially explaining the lack of differences. These findings agree with those previously observed by Safdar et al., who also found that the association between the ApoE ε4 genotype and COVID-19 severity is not related to medical comorbidities [5].

The current and previous findings [10,11] obtained from the same sample of individuals who had been hospitalized due to COVID-19 could suggest that specific polymorphisms associated with COVID-19, such as ACE2, ACE1, TMPRSS2, and ApoE, are not related to the presence of post-COVID symptomatology. However, these findings do not exclude a potential role of other genetic markers or gene–environment interactions in the development of long COVID. For instance, it is possible that other types of genetic analysis, such as DNA methylation, could reveal potential associations between different genes and long COVID. Thus, future studies investigating other genetic markers would further contribute to the understanding of the genetic basis of long COVID.

Some limitations of the current study should be recognized. First, only previously hospitalized COVID-19 survivors were included, accordingly, the role of the ApoE gene in non-hospitalized patients should be also investigated in future studies. Second, we recognize that we included a limited sample size, and it is possible that larger samples can identify genotype differences; accordingly, our study should be considered exploratory. Accordingly, population-based cohort studies including a whole-genome SNP analysis will help to validate or refute our results and identify other genes potentially related to long COVID symptomatology. Finally, post-COVID symptoms were self-reported at the time of the interview, which introduces the potential for recall bias and subjective interpretation of symptoms by the participants. Current evidence points to variability in symptoms and their duration among COVID-19 survivors [18]; accordingly, studies using repeated measures assessing the presence of symptoms at different moments would help to confirm our results. 

## 5. Conclusions

This secondary analysis revealed that the ApoE ε4 genotype, albeit previously associated with a higher risk of SARS-CoV-2 infection and COVID-19 severity, did not appear to predispose for the presence of long COVID symptoms in a cohort of previously hospitalized COVID-19 survivors. However, these data should not be extrapolated to non-hospitalized COVID-19 survivors and future studies should investigate the potential association of the ApoE ε4 genotype in a more heterogeneous population of individuals who were infected with SARS-CoV-2.

## Figures and Tables

**Table 1:** Pre-Infection data and post-COVID symptoms according to the ApoE Genotype (*n* = 287).

	ApoE ε2 (*n* = 25)	ApoE ε3 (*n* = 204)	ApoE ε4 (*n* = 58)	*p*-Value
Age, mean (SD), years *	61.2 (11.0)	57.0 (12.6)	53.4 (13.8)	0.03
Females/males (*n*)	13/12	101/103	29/29	0.986
Weight, mean (SD), kg	73.7 (10.8)	81.8 (17.0)	80.5 (17.5)	0.07
Height, mean (SD), cm *	165.5 (9.0)	167 (9.5)	168 (8.5)	0.636
Number of comorbidities, mean (SD)	1.0 (0.75)	1.3 (1.0)	1.3 (1.1)	0.298
Medical comorbidities, *n* (%)				
Hypertension	12 (48.0%)	69 (33.8%)	19 (32.75%)	0.503
Diabetes	3 (12.0%)	10 (9.8%)	7 (12.1%)	0.867
Cardiovascular Diseases	0 (0.0%)	17 (8.3%)	4 (6.9%)	0.344
Asthma	0 (0.0%)	24 (11.8%)	7 (12.1%)	0.227
Obesity	2 (8.0%)	67 (32.8%)	20 (34.5%)	0.09
Chronic Obstructive Pulmonary Disease	0 (0.0%)	5 (2.5%)	1 (1.7%)	0.709
Number of post-COVID symptoms, mean (SD)	2.8 (2.1)	3.0 (1.9)	3.2 (1.9)	0.694
Post-COVID symptoms, *n* (%)				
Fatigue	13 (52.0%)	127 (62.25%)	41 (70.7%)	0.594
Dyspnoea at exertion	14 (56.0%)	138 (67.65%)	44 (75.85%)	0.599
Memory Loss	6 (24.0%)	62 (30.4%)	23 (39.7%)	0.421
Hair Loss	7 (28.0%)	51 (25.0%)	19 (32.75%)	0.598
Concentration Loss	2 (8.0%)	32 (15.7%)	10 (17.25%)	0.597
Cognitive Blunting—Brain Fog	2 (8.0%)	30 (14.7%)	8 (13.8%)	0.697
Dyspnoea at rest	1 (4.0%)	27 (13.25%)	12 (20.7%)	0.154
Ocular Disorders	5 (20.0%)	28 (13.7%)	8 (13.8%)	0.731
Anosmia/Hyposmia	1 (4.0%)	23 (11.3%)	5 (8.6%)	0.515
Skin Rashes	12 (11.0%)	28 (13.7%)	8 (13.8%)	0.782
Gastrointestinal Disorders *	6 (24.0%)	18 (8.8%)	3 (5.2%)	0.03
Ageusia/Hypogeusia	2 (8.0%)	13 (6.4%)	5 (8.6%)	0.831
Months with post-COVID symptoms, mean (SD)	18.0 (4.5)	17.6 (5.3)	18.0 (5.1)	0.460
Days in hospital, mean (SD)	10.0 (10.7)	8.2 (8.6)	7.6 (9.0)	0.563

* Statistical significant differences among groups by ApoE genotype.

## Data Availability

All data derived from this study are presented in the text.

## References

[1] Glotov O.S., Chernov A.N., Scherbak S.G., Baranov V.S. (2021). Genetic risk factors for the development of COVID-19 coronavirus infection. Russ. J. Genet..

[2] Ferreira de Araújo J.L., Menezes D., Saraiva-Duarte J.M., de Lima F.L., Santana de Aguiar R., Pedra de Souza R. (2022). Systematic review of host genetic association with Covid-19 prognosis and susceptibility: What have we learned in 2020?. Rev. Med. Virol..

[3] Zhao J., Lu W., Ren Y., Fu Y., Martens Y.A., Shue F., Davis M.D., Wang X., Chen K., Li F. (2021). Apolipoprotein E regulates lipid metabolism and alpha-synuclein pathology in human iPSC-derived cerebral organoids. Acta Neuropathol..

[4] Dieter C., Brondani L.A., Leitão C.B., Gerchman F., Lemos N.E., Crispim D. (2022). Genetic polymorphisms associated with susceptibility to COVID-19 disease and severity: A systematic review and meta-analysis. PLoS ONE.

[5] Safdari Lord J., Soltani Rezaiezadeh J., Yekaninejad M.S., Izadi P. (2022). The association of APOE genotype with COVID-19 disease severity. Sci. Rep..

[6] Gkouskou K., Vasilogiannakopoulou T., Andreakos E., Davanos N., Gazouli M., Sanoudou D., Eliopoulos A.G. (2021). COVID-19 enters the expanding network of apolipoprotein E4-related pathologies. Redox Biol..

[7] Chen F., Chen Y., Ke Q., Wang Y., Gong Z., Chen X., Cai Y., Li S., Sun Y., Peng X. (2023). ApoE4 associated with severe COVID-19 outcomes via downregulation of ACE2 and imbalanced RAS pathway. J. Transl. Med..

[8] Fernández-de-las-Peñas C. (2022). Long COVID: Current definition. Infection.

[9] Soriano J.B., Murthy S., Marshall J.C., Relan P., Diaz J.V. (2022). WHO Clinical Case Definition Working Group on Post-COVID-19 Condition. A clinical case definition of post-COVID-19 condition by a Delphi consensus. Lancet Infect. Dis..

[10] Fernández-de-Las-Peñas C., Arendt-Nielsen L., Díaz-Gil G., Gómez-Esquer F., Gil-Crujera A., Gómez-Sánchez S.M., Ambite-Quesada S., Palomar-Gallego M.A., Pellicer-Valero O.J., Giordano R. (2022). Genetic association between ACE2 (rs2285666 and rs2074192) and TMPRSS2 (rs12329760 and rs2070788) polymorphisms with post-COVID symptoms in previously hospitalized COVID-19 survivors. Genes.

[11] Fernández-de-Las-Peñas C., Arendt-Nielsen L., Díaz-Gil G., Gil-Crujera A., Gómez-Sánchez S.M., Ambite-Quesada S., Palomar-Gallego M.A., Pellicer-Valero O.J., Giordano R. (2023). ACE1 rs1799752 polymorphism is not associated with long-COVID symptomatology in previously hospitalized COVID-19 survivors. J. Infect..

[12] Tsampasian V., Elghazaly H., Chattopadhyay R., Debski M., Naing T.K.P., Garg P., Clark A., Ntatsaki E., Vassiliou V.S. (2023). Risk factors associated with post-COVID-19 condition: A systematic review and meta-analysis. JAMA Intern. Med..

[13] Smatti M.K., Al-Sarraj Y.A., Albagha O., Yassine H.M. (2020). Host genetic variants potentially associated with SARS-CoV-2, A multi-population analysis. Front. Genet..

[14] Lumsden A.L., Mulugeta A., Zhou A., Hyppönen E. (2020). Apolipoprotein E (APOE) genotype-associated disease risks: A phenome-wide, registry-based, case-control study utilising the UK Biobank. EBioMedicine.

[15] Notarte K.I., de Oliveira M.H.S., Peligro P.J., Velasco J.V., Macaranas I., Ver A.T., Pangilinan F.C., Pastrana A., Goldrich N., Kavteladze D. (2022). Age, sex and previous comorbidities as risk factors not associated with SARS-CoV-2 infection for long COVID-19, A systematic review and meta-analysis. J. Clin. Med..

[16] Fernández-de-las-Peñas C., Torres-Macho J., Velasco-Arribas M., Plaza-Canteli S., Arias-Navalón J.A., Hernández-Barrera V., Guijarro C. (2022). Preexisting hypertension is associated with a greater number of long-term post-COVID symptoms and poor sleep quality: A case-control study. J. Hum. Hypertens..

[17] Fernández-de-las-Peñas C., Guijarro C., Torres-Macho J., Velasco-Arribas M., Plaza-Canteli S., Hernández-Barrera V., Arias-Navalón J.A. (2021). Diabetes and the risk of long-term post-COVID symptoms. Diabetes.

[18] Fernández-de-las-Peñas C. (2022). Are patients exhibiting post-coronavirus disease (COVID) symptoms at 12 months the same at 5 or 9 months? The fluctuating nature of post-COVID. Clin. Infect. Dis..

